# Changes of Cadmium Storage Forms and Isotope Ratios in Rice During Grain Filling

**DOI:** 10.3389/fpls.2021.645150

**Published:** 2021-04-29

**Authors:** Matthias Wiggenhauser, Anne-Marie Aucour, Philippe Telouk, Hester Blommaert, Géraldine Sarret

**Affiliations:** ^1^Institute of Agricultural Sciences, Department of Environmental Systems Science, Eidgenössische Technische Hochschule Zürich, Zurich, Switzerland; ^2^ISTerre, Université Grenoble Alpes, Université Savoie Mont Blanc, Centre National de la Recherche Scientifique, Institut de Recherche pour le Développement, Institut Français des Sciences et Technologies des Transports, de l’Aménagement et des Réseaux, Grenoble, France; ^3^Laboratoire de Geologie de Lyon, Ecole Normale Supérieure de Lyon, Université Lyon 1, Université de Lyon, Centre National de la Recherche Scientifique, Lyon, France

**Keywords:** cadmium, rice, soil, stable isotope fractionation, X-ray absorption spectroscopy, speciation, remobilization and storage

## Abstract

Rice poses a major source of the toxic contaminant cadmium (Cd) for humans. Here, we elucidated the role of Cd storage forms (i.e., the chemical Cd speciation) on the dynamics of Cd within rice. In a pot trial, we grew rice on a Cd-contaminated soil in upland conditions and sampled roots and shoots parts at flowering and maturity. Cd concentrations, isotope ratios, Cd speciation (X-ray absorption spectroscopy), and micronutrient concentrations were analyzed. During grain filling, Cd and preferentially light Cd isotopes were strongly retained in roots where the Cd storage form did not change (Cd bound to thiols, Cd–S = 100%). In the same period, no net change of Cd mass occurred in roots and shoots, and the shoots became enriched in heavy isotopes (Δ^114/110^Cd_*maturity–flowering*_ = 0.14 ± 0.04‰). These results are consistent with a sequestration of Cd in root vacuoles that includes strong binding of Cd to thiol containing ligands that favor light isotopes, with a small fraction of Cd strongly enriched in heavy isotopes being transferred to shoots during grain filling. The Cd speciation in the shoots changed from predominantly Cd–S (72%) to Cd bound to O ligands (Cd–O, 80%) during grain filling. Cd–O may represent Cd binding to organic acids in vacuoles and/or binding to cell walls in the apoplast. Despite this change of ligands, which was attributed to plant senescence, Cd was largely immobile in the shoots since only 0.77% of Cd in the shoots were transferred into the grains. Thus, both storage forms (Cd–S and Cd–O) contributed to the retention of Cd in the straw. Cd was mainly bound to S in nodes I and grains (Cd–S > 84%), and these organs were strongly enriched in heavy isotopes compared to straw (Δ^114/110^Cd_*grains/nodes–*__*straw*_ = 0.66–0.72‰) and flag leaves (Δ^114/110^Cd_*grains/nodes–flag leaves*_ = 0.49–0.52‰). Hence, xylem to phloem transfer in the node favors heavy isotopes, and the Cd–S form may persist during the transfer of Cd from node to grain. This study highlights the importance of Cd storage forms during its journey to grain and potentially into the food chain.

## Introduction

Cadmium (Cd) is a nonessential element for most biota (Sigel et al., 2013). It has a long biological half-life time in the human body, it is considered as class 1 carcinogen (WHO/IARC, 2020), and its accumulation impairs organs such as the kidney (Godt et al., 2006; Fransson et al., 2014). Due to its accumulative behavior, concentrations of 0.2–0.4 mg (kg DW)^–1^ in staple crops such as wheat and rice are considered as harmful for humans (FAO, 2009), while plants can cope with Cd concentrations up to 10 mg (kg DW)^–1^ in their aboveground tissues (White, 2012). Many agricultural soils are Cd contaminated [>1 mg Cd (kg soil)^–1^] through anthropogenic Cd emissions or Cd that occurs naturally in soil bedrocks (Smolders and Mertens, 2013; Zhang et al., 2015; Tóth et al., 2016; Liu et al., 2017). Thus, many crop-producing areas face the challenge of providing crops with low Cd contents. In several Asian countries, rice is the major Cd source for humans (Al-Rmalli et al., 2012; Chunhabundit, 2016; Song et al., 2017). The combination of rice as major food source, subsistence farming, and elevated Cd concentrations in rice can regionally lead to daily Cd intake that exceeds critical limits given by international authorities (Meharg et al., 2013; Wang P. et al., 2019). To apply agricultural measures that reduce Cd in staple crops, the understanding of processes that control the mobility of Cd in soil–plant systems is crucial.

On its pathway from soil to grain, Cd has to cross several barriers. It is taken up by rice with root membrane transporters that are thought to target cationic nutrients such as manganese (Mn), iron (Fe), zinc (Zn), and calcium (Ca) (Ishimaru et al., 2006; Nakanishi et al., 2006; Lee and An, 2009; Takahashi et al., 2011; Sasaki et al., 2012; Chen et al., 2018). In the roots, Cd is partially separated from nutrients by being transported into the vacuoles by metal pumps and by binding Cd to stable sulfur (S) containing low molecular weight organic ligands (Ueno et al., 2010; Cao et al., 2018; Cai et al., 2019; Wiggenhauser et al., 2021). The fraction of Cd in the root that is still mobile can be exported from the root cytosol via xylem into the shoot by metal pumps (Nocito et al., 2011; Satoh-Nagasawa et al., 2012). To be transported into the grain, Cd is either (i) directly taken up from the roots and transported into the grains or (ii) remobilized from plant organs that stored mineral nutrients during vegetative plant growth (Maillard et al., 2015; Wang et al., 2018; Yan et al., 2018, 2019). The remobilization of nutrients and Cd from vegetative tissues to grains requires a network of well-synchronized processes. This includes degradation of cell components and organic molecules in stems and leaves, membrane transporters, and the vascular tissues xylem and phloem for long distance transport (Pottier et al., 2014; Etienne et al., 2018). In rice, the nodes play a crucial role for the transfer of nutrients and Cd by connecting xylem and phloem and regulating the intervascular transfer of Cd and nutrients facilitated by membrane transporters (Uraguchi et al., 2011; Yamaji et al., 2013; Yamaji and Ma, 2014; Hao et al., 2018; Tan et al., 2019). Besides membrane transporters that act as gate keepers in metal trafficking within the shoot, Cd storage forms (i.e., the chemical Cd species) may also play an important role in controlling the mobility of metals in the shoot during grain filling.

Only a few studies have determined the Cd storage forms in rice. Synchrotron X-ray absorption spectroscopy (XAS) revealed that more than 90% of Cd was bound to S groups in roots that grew on Cd-contaminated conditions (Yamaoka et al., 2010; Yan et al., 2016; Wiggenhauser et al., 2021). In stems, leaves, and nodes, Cd bound to S donors (Cd–S) was found in all cases, but unlike the root, Cd bound to oxygen (O) donors (Cd–O) was, in some cases, the major binding form of Cd to the shoots (up to 94%) (Yamaoka et al., 2010; Yamaguchi et al., 2012; Yan et al., 2016). Little is known about Cd speciation in rice grains. Size exclusion chromatography–inductively coupled plasma mass spectrometry (ICP-MS) techniques (Wei et al., 2017) indicated that the major fraction of Cd was stored with S-rich protein fractions in rice. Recent X-ray absorption spectroscopy (XAS) studies for rice and wheat showed that Cd–S can be an important storage form in grains (27–92%, Gu et al., 2020; Yan et al., 2020). To date, no study reported Cd storage forms in rice roots, shoots, and grains and how these storage forms change at different growth stages.

An approach to study the role of Cd storage forms in plants on the dynamics within rice could be the combination of Cd mass balances, X-ray absorption near edge structure (XANES) spectroscopy, and Cd isotope fractionation (Ryan et al., 2013; Aucour et al., 2015, 2017; Zelano et al., 2020; Wiggenhauser et al., 2021). The XAS measurements deliver snapshots of the speciation of metals, i.e., of the metal storage forms in plants while Cd mass balances and Cd isotopes can provide information on the dynamics of metals in soil–plant systems. Metal stable isotope fractionation in plants can be induced by processes such as complexation, chelation, diffusion, and membrane transport (Caldelas and Weiss, 2017). This isotope fractionation then leads to small but distinguishable isotope ratios in different plant tissues. For nonredox-sensitive elements such as Cd and Zn, membrane transport (John et al., 2007; Köbberich and Vance, 2017; Moore et al., 2020) and binding of the metals to organic and inorganic ligands are key processes for isotope fractionation (Jouvin et al., 2009; Fujii et al., 2014; Yang et al., 2015; Marković et al., 2017; Guinoiseau et al., 2018). Experimentally and theoretical determined isotope fractionation factors revealed that Cd bound to S is lighter than hydrated Cd and hydrated Cd is lighter than Cd bound to O and N groups of inorganic and organic ligands (Yang et al., 2015; Guinoiseau et al., 2018; Zhao et al., 2021). Thus, Cd isotopes can potentially serve as speciation marker for thermodynamically controlled Cd binding processes (Horner et al., 2013; Wiggenhauser et al., 2016). In addition, processes that control the mobility of metals in living organisms can be also partly kinetically controlled through, e.g., fast and unidirectional membrane transport (Köbberich and Vance, 2017). Such a kinetically controlled Cd isotope fractionation may occur during Cd uptake into plants and leads to an enrichment of light isotopes compared to soil solution or nutrient solution in hydroponics (Wei et al., 2016; Imseng et al., 2019; Moore et al., 2020; Zhang et al., 2021).

The objective of this study was to determine the relation of Cd storage forms in rice on the dynamics of Cd and its isotopes during grain filling. We expected that Cd storage forms in roots and shoots alter during grain filling and partly control the pathways of Cd and its isotopes in rice. To this end, we grew rice at upland conditions and harvested the rice at flowering and maturity. For the first time, Cd concentration, isotopes, and speciation as well as concentration of metallic micronutrients were measured at different growth stages and in several rice parts including nodes and rice grains. Cd isotopes were used to follow Cd remobilization pathways during grain filling, in relation to Cd storage forms. Finally, these data were set into context with Cd regulation and detoxification in rice.

## Materials and Methods

### Pot Trial

Rice (*Oryza sativa* L., cv Taichung-65) was grown at upland conditions in a growth chamber. Four replicates were harvested at flowering stage and four replicates at maturity. For this study, the growth period until flowering is defined as vegetative growth period. We defined the period between flowering and maturity as grain filling period. Part of the data presented for the flowering stage was already published in Wiggenhauser et al. (2021); details are given in the legends and footnotes of the figures and tables, respectively.

The soil was sampled from a rice production area in Northern Italy, Briona. The soil texture consists of >50% sand, the cation exchange capacity was 46.2 ± 5.2 [mmolc (kg soil)^–1^], the maximal water holding capacity was 0.60 [L water (kg soil)^–1^], and the soil pH was at 4.9 (Wiggenhauser et al., 2021). About 15 portions of soil each containing ∼15 kg of fresh soil were taken from randomly distributed spots from the rice field in autumn 2017. The soil was then dried and sieved (5 mm) and further homogenized in a concrete mixer. Before 1.5 kg of dry soil was added to pots, 22.5 mg Cd was added to the soils in liquid form as Cd nitrate [Cd(NO_3_)_2_], and the soil was then thoroughly mixed. They soils were aged for 21 days to equilibrate the added Cd with the soil (Ren et al., 2016). For soil aging, the soils were kept at room temperature in the dark, and water levels were kept between 40 and 80% of the maximum water holding capacity. Twenty-three days before the seedlings were planted into the soils, the soils were fertilized. To this end, the soils of each individual pot were transferred into a bowl; 160 mg of N, 50 mg phosphorus (P), 83 mg potassium (K), 12 mg Ca, 20 mg magnesium (Mg), 44 mg S, and 5 mg Zn per kilogram of soil were added to each soil in liquid form, and the soil and fertilizer were thoroughly mixed. Then, a rhizon pore water sampler (SDEC, France) was installed when the fertilized soils were transferred back into the pots. For the full plant growth period, pH and redox potential (Eh) were measured at minimum biweekly. The Cd spiked and fertilized soils were then equilibrated until the rice seedlings were planted into the pots. Fifty-four days after sowing (DAS), an additional fertilization was added to the soil (80 mg N, 50 mg P, 83 mg K, 8.2 mg S per kilogram of soil).

Rice seedlings were germinated in wet vermiculite in the dark. For nursery, the seedlings were transferred into a nonaerated hydroponic system that contained one-half strength Kimura B nutrient solution (pH 5.6) from 7 to 25 DAS (Shao et al., 2017). No Cd was added to the hydroponic nutrient solution. After 25 days, the plants were transferred to the preconditioned soils. For nursery and plant growth in the pots, the rice plants were kept in a growth chamber (Aralab, France). The temperature was set to 30°C during the day and 25°C at night. The humidity was set to 70%, and the light three stages of light intensity (0, 300, and 600 μM m^–2^ s^–1^) were used to mimic a photoperiod of 11 h/day (Köhl, 2015; Wiggenhauser et al., 2021). The light intensity was frequently measured at a height that represented one-half of the rice shoot, and the light intensity was continuously adjusted to maintain the light intensity during the entire plant growth period. The water level of the pots was kept between 40 and 90% of the total water holding capacity.

### Harvest and Sample Preparation

Soil and rice were sampled at flowering (94 DAS) and maturity (126 DAS). We defined “flowering” when three tillers were having flowers, which is equal to growth stage R4 according to Counce et al. (2000). Maturity was defined since the rice grains reached dough stage, which is equal to R7–R8 in Counce et al. (2000). The water content of the rice grains were <28% at harvest. Rice shoots were cut 1 cm above the soil surface, rinsed using deionized water (>18.2 MΩ), and gently dried with paper towels. The shoot was then dissected into different parts using a clean scissor (graphical support in Supplementary Figure 1). At flowering, the nodes were separated from the remaining shoot (i.e., whole shoot without nodes). At maturity, the shoot was dissected into nodes, straw, and upper shoot. The latter was further dissected into flag leaves, panicle (rachis and spikelets), and grains. For both growth stages, the nodes sampled are defined as “node I” that is located below the panicle node and connects straw, flag leaf, and upper shoot (Yamaji and Ma, 2014). At maturity, the straw represents all stems and leaves below node I, and the shoot dry weight (DW) and elemental mass of the shoot was calculated as the sum of all shoot parts and the elemental concentration and Cd isotope ratios as weighted mean according to Eq. (2). After dissection, the fresh weight was recorded, and the shoot parts were dissected into small pieces of about 1 cm, transferred into a clean beaker, and thoroughly homogenized with a clean plastic spoon.

Shoot, straw, and flag leaves were split into two aliquots. A first aliquot was prepared for speciation measurements. To this end, the aliquots were frozen in liquid nitrogen (N_2liq_) and milled using a cryogrinder (Pulverisette 23, Fritsch). The milled samples were transferred into cryotubes and immediately stored at −80°C in a freezer. A second aliquot was prepared for concentration and isotope analysis. To this end, the fresh weight was recorded prior to drying in an oven at 55°C. When the samples were dry, the dry weight was recorded, and the samples were milled using a planetary mill (Pulverisette 7, Fritsch) equipped with agate cups and balls.

The nodes and the grains were processed in a slightly different manner. At both growth stages, the first node below the panicle node was separated from the shoots. The DW of the nodes was small (<0.30 g); thus, the nodes of two rice replicates were merged, which resulted in two experimental replicates for the nodes for speciation, concentration, and isotope analysis. To assure that sufficient Cd was available for concentration and isotope analysis, the nodes selected for concentration and isotope analysis of the shoot were not milled to avoid losses of dry matter during milling. Instead, the entire aliquots of the nodes were digested (see next section). For the grains, the spikes (panicle and grains) were separated from the remaining shoot and dried with paper towel. From each tiller, six randomly chosen grains were removed from the spikes and milled using the cryogrinder as described above. The fresh weight of the remaining spikes was reported prior to oven drying at 50°C. After drying, the dry weight was reported, and the grains were then separated from the spikes. The remaining part of the spike was defined as panicle (rachis and spikelets). Finally, dry weights from grains and panicle were taken prior to milling with a planetary mill as described above.

At flowering and maturity, the soil was gently separated from the roots by hand. The soil that still stuck on the roots was removed by dipping and shaking the roots in deionized water that immediately turned into a soil suspension. The roots were then gently dried using a paper towel and then split into two aliquots for speciation and concentration/isotope analysis as described in detail above for the shoot. The roots were immediately stored at −80°C. An aliquot of fresh soil (equivalent of about 3 g of dry soil) was taken after the plant harvest to determine the calcium nitrate [Ca(NO_3_)_2_] extractable Cd fraction from the soil (Gray et al., 1999; Wiggenhauser et al., 2016). About 20 g of the soils from the pots were sampled and air dried at 55°C to determine the water content of the soil at harvest.

### Sample Extraction, Digestion, and Purification

For all extractions, digestions, and matrix separation procedures, distilled nitric (HNO_3_) and hydrochloric acid (HCl) were used. In addition, commercially available hydrofluoric acid (HF, 47–51% trace metal grade, Fisher Chemical) and hydrogen peroxide (H_2_O_2_, 30%, Suprapur, Merck) were used for sample digestion, while “analytical” grade salts were used for Cd soil spiking and soil fertilization. All laboratory work was conducted in the clean lab facilities of ISTerre Grenoble. For plant watering and laboratory work, deionized water was used (>18.2 MΩ). Samples were digested and evaporated in PFA beakers. Before analysis, samples were stored in polypropylene tubes (Metal-Free Centrifuge Tubes, Polypropylene, VWR).

To determine the Ca(NO_3_)_2_ extractable Cd fraction of the soil, 30 ml 0.05 M Ca(NO_3_)_2_ was added to an equivalent of 3 g of dry soil; the samples were shaken for 16 h, centrifuged (3500 *g*) for 20 min, and then filtered (Rotilabo KH55.1 0.45 μm, Carl Roth). Prior to storage in the fridge, 1 ml 14 M distilled HNO_3_ was added to the samples. One hundred milligrams of soil powder was digested in 6 ml distilled 14 M HNO_3_ + 2 ml HF. For root and shoot samples, 200 mg of powder were predigested for >12 h in 7 M HNO_3_, evaporated, and digested in 6 ml 14 M HNO_3_ + 2.5 ml H_2_O_2_ + 0.5 ml HF for >48 h (Wiggenhauser et al., 2021).

After digestion, the samples were purified for isotope analysis by removing matrix elements from the sample using resin anion exchange chromatography. To this end, the samples were evaporated, refluxed in 7 M HCl for >12 h, evaporated, and then resolubilized in 2 M HCl. The sample was then split into two aliquots, of which a small aliquot was used to measure the Cd concentration in unpurified samples, while the large aliquot of the sample was purified for isotope analyses. For the purification, we slightly adapted a method that was developed for environmental samples that are Cd contaminated (Cloquet et al., 2005; Wei et al., 2015). Polypropylene columns (Poly-Prep, Bio-Rad) were filled with 2 ml resin (AG-MP-1). After cleaning the resin using 30 ml 0.5 M HNO_3_, the resin was preconditioned using 4 ml H_2_O followed by 4 ml 2 M HCl. The sample that was dissolved in 4 ml 2 M HCl was then loaded on the resin. Matrix elements were subsequently eluted from the column using 8 ml 2 M HCl, 12 ml 0.3 M HCl, and 14 ml 0.012 M HCl. Finally, Cd was eluted into Teflon beakers using 22 ml 0.0012 ml HCl.

For speciation analysis, the cryomilled and frozen samples were pressed to pellets. To this end, the sample powder was transferred into a precooled die set (plunged in liquid N_2_), quickly transferred into a hydraulic press, and back into a container that contained liquid N to remove the pressed pellet from the die set. The pressed pellets had a diameter of 5 mm and a thickness of 1–4 mm. During storage, transport to the synchrotron facilities, and mounting samples on the sample holder at the synchrotron facilities, samples were kept in −80°C or liquid N to ensure no changes in Cd speciation.

### Concentration and Isotope Analysis

Concentrations of Cd, Zn, Cu, Mn, and Ni were measured using a single collector quadrupole inductively coupled plasma mass spectrometry (ICP-MS) (ICP-MS, X-Series II, Thermo Fisher). Cd isotopes were measured using a high-resolution multicollector ICP mass spectrometer (MC-ICPMS, Neptune Plus, Thermo Scientific). The same cup configuration was chosen for the MC-ICPMS as reported in Pallavicini et al. (2014). To correct for instrumentally induced mass bias, sample bracketing and Ag doping was applied using a Cd/Ag concentration ratio of 2:1 (Wombacher et al., 2003; Cloquet et al., 2005). Isotope ratios were measured at Cd concentrations of either 100 or 200 μg L^–1^.

### Cd Speciation Analysis

Cadmium solid speciation was measured using Cd K-edge X-ray absorption spectroscopy in the synchrotron beamlines SAMBA (Soleil, Paris), CLAESS (ALBA, Barcelona), and PETRA III P64 (DESY, Hamburg). For all samples, the X-ray absorption near edge structure (XANES) and, if possible, the extended X-ray absorption fine structure (EXAFS) part were analyzed. All samples were recorded at 20 K using a He cryostat in fluorescence mode. In all beamlines, a Cd or Ag foil was recorded to calibrate the inflection point of the edge at 26,711 and 25,514 eV, respectively. With XANES, the proportion of Cd bound to O (Cd–O) and to S (Cd–S) donor atoms can be determined. Depending on the concentration of the samples, we additionally fitted the spectra with EXAFS to provide more robust results than with XANES only. To record EXAFS part of the spectra in a sufficient quality depends on the Cd concentration in the sample, on the duration of spectra recording time, and on the sensitivity of the beamline. In this study, Cd dry weight concentrations were highest in roots (>420 μg g^–1^); for these samples, EXAFS spectra were used for linear combination fits (LCF, see next paragraph). All shoot samples were <54 μg g^–1^, and EXAFS was not in all cases used for LCF. The lowest Cd concentration was found in the grains (0.98 μg g^–1^). At the spectroscopy applied to material based on absorption (SAMBA) beamline, we managed to record a spectrum of the XANES part of grains within acquisition time of ∼15 h.

The recorded spectra were normalized and then treated by LCFs using XAS software [Athena, Demeter, Ravel and Newville (2005)]. To this end, a database of Cd reference spectra was employed (Huguet et al., 2012, 2015; Fulda et al., 2013), and the LCFs were then grouped as “Cd–O” (Cd bound to COOH/OH groups of organic matter or Cd sorbed to minerals) and “Cd–S,” which represents Cd bound to thiols (R-SH). Crystalline CdS, previously identified in the same pot experiment but in flooded conditions (Wiggenhauser et al., 2021), was absent in the present case of soil in wet conditions.

### Data Quality Assessment

The quality of the digestion and purification procedure was frequently tested and revealed robust results as reported in detail in Wiggenhauser et al. (2021). Briefly, the purification procedure gained a Cd recovery of 90–102% (mean = 97%). Cadmium in the procedural blanks was <0.12% of Cd in the samples. The Cd/Sn mass ratios of the purified samples were >1000. Overall, we can exclude artifacts on isotope ratios that were induced during sample processing. Metal concentration analysis was tested by frequently measuring internal and certified standard material. The Cd recovery of certified standards ranged between 92 and 107% (Supplementary Table 1). For all other metals, quality tests revealed a high precision and a sufficient accuracy with a recovery of 88–117%.

Cadmium isotope ratios were measured in soil (NIST SRM 2711) and plant samples (white cabbage, BCR-679) that are certified for Cd concentration. In the soil sample, Cd isotopes were measured previously, which allowed for interlaboratory comparison of the results (Cloquet et al., 2005; Pallavicini et al., 2014; Liu et al., 2019). Furthermore, Cd isotope ratios of liquid reference sample (Münster Cd) were measured (Abouchami et al., 2013). The analytical precision of the isotope measurements expressed as two times standard deviation (2sd) was δ^114/110^Cd = 0.04‰ of *n* = 5 samples. The precision of process replicates of soil and plant samples was 2sd = 0.04‰ of *n* = 12 samples (more details in Supplementary Table 1). These are typical precisions for Cd isotope analysis (Rehkämper et al., 2012). In addition, mean isotope ratios agreed well with previously published values, which proves the accuracy of the chosen method (see details in Wiggenhauser et al., 2021).

For selected samples, XAS spectra were recorded at different beamlines, as well as experimental replicates were measured. The analytical precision and LCF for plant samples ranged from sd = 0–1% (*n* = 2 analytical replicates), and for soil samples, sd = 14% (*n* = 2 analytical replicates). The variation in individual plants ranged from 0 to 1.04% (*n* = 2 experimental replicates) and for soils from 7 to 15% (*n* = 2 experimental replicates). These results confirm the “rule of thumb” that the precision for XAS speciation measurements is in average approximately ±10% (Castillo-Michel et al., 2017).

### Calculations

Isotope ratios of ^114^Cd/^110^Cd of samples are expressed relative to the ^114^Cd/^110^Cd isotope ratio of isotope reference material NIST_3108_ as δ in per mill,

(1) δ114/110Cd=[(C114d/110Cd)sample(C114d/110Cd)NIST.3108-1]×1000

The Cd isotope ratio of the entire plants at both growth stages (root + shoot) or the entire shoots at maturity (all shoot parts at maturity) were calculated as a weighted arithmetic mean,

(2) $$\delta^{114/110}Cd_{wholeplant}=\frac{\sum_{i}m_{i}c_{i}\delta^{114/110}Cd_{i}}{\sum m_{i}c_{i}}$$

where “*m*” denotes DW (g); “*c*_*i*_” the Cd concentration (μg g^–1^) of, e.g., the root, shoot, and grains (expressed as “i”); and δ the corresponding Cd isotope ratio. The nodes were not taken into account since their DW was compared to the total dry weight of the shoot negligible. Similar weighted mean calculations were applied to calculate average concentrations in entire plants and shoots by using the DW as weighing factor.

Apparent Cd isotope fractionation was expressed as “Δ” by subtracting Cd isotope ratios from each other,

(3) $$\Delta^{114/110}Cd_{A-B}=\delta^{114/110}Cd_{A}-\delta^{114/110}Cd_{B}$$

where “A” and “B” are the isotope ratios of compartments of the system, e.g., grain and straw.

The difference in metal concentration, metal mass, and Cd isotope ratios during grain filling were calculated as

(4) $$mean_{grain.filling}=mean_{maturity}-mean_{flowering}$$

where “mean” denotes the mean values of experimental replicates. The error bars were calculated as

(5) $$sd_{total}=\sqrt{{\left(sd_{flowering}\right)}^{2}+{\left(sd_{maturity}\right)}^{2}}$$

where sd denotes the standard deviation of experimental replicates.

### Statistics

In this study, we distinguished between analytical precision (same sample measured several times), process replicates (same sample processed and measured several times), and experimental replicates (e.g., replicates of individually grown plant samples that were processed and analyzed individually, Dürr-Auster et al., 2019). For metal concentrations and Cd isotope ratios, *n* = 4 experimental replicates were measured. Significant differences of the mean of experimental replicates were tested with either ANOVA and Welch’s *t*-test or their nonparametric equivalents Kruskal–Wallis test and Wilcoxon rank sum test, respectively. ANOVA [followed by a Tukey honestly significant difference (HSD) test] was applied to compare, e.g., the mean isotope ratio of different plant organs (*n* > 2) at one growth stage, if equality of variances was given (determined using a Levene test and visual inspection), and the distribution of residuals were normally distributed (visual inspection). If these prerequisites were not fulfilled, the data were log_10_, square root, or 1/x transformed. If the prerequisites for an ANOVA were still not fulfilled, a one factorial Kruskal–Wallis (followed by Conover–Iman test) was applied. A Welch’s *t*-test was applied to compare, e.g., the isotope ratio of the same plant organ at flowering and maturity. If the data were not normally distributed (Lilliefors test), a Wilcoxon rank sum test was applied. For all tests, mean values were considered as significantly different from each other if the *p* < 0.05. Statistical tests and figure were computed using the statistical software R (v.3.4.4; R Foundation, Vienna).

Due to limited DW, it was not possible to measure Cd concentration, isotope ratios, and speciation in *n* = 4 experimental replicates in the nodes. Thus, samples were merged to *n* = 2 experimental replicates. The variation in Cd concentration, Cd mass, and Cd isotope ratios were expressed as 2sd. The mean values were considered as significantly different from each other when the error bars did not overlap. Due to limited synchrotron beamtime, we recorded *n* = 1–3 replicates (Supplementary Table 6). Based on analytical and processing replicates, we considered the Cd speciation as significantly different from each other when they differed by >20% (see the section “Data Quality Assessment”).

## Results

### Cd in Soil and Rice

**FIGURE 1:**
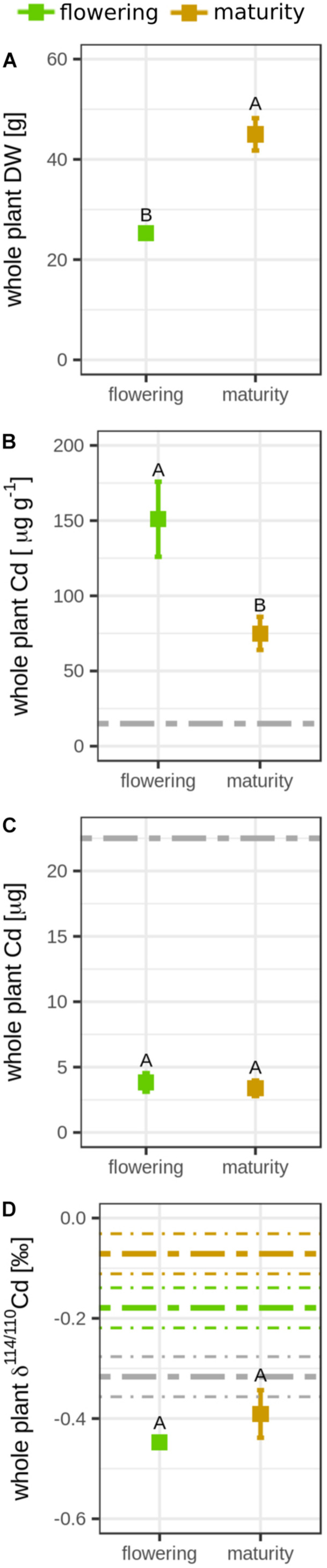
Mean **(A)** dry weight (DW), **(B)** cadmium (Cd) concentration, **(C)** Cd mass, and **(D)** Cd isotope ratio of the whole rice plants that were harvested at flowering and maturity. Error bars for plants represent the standard deviation of *n* = 4 experimental replicates. Letters denote significant differences of the mean that were determined by either **(B,C)** a Welch’s *t-*test or **(A,D)** a Wilcoxon rank sum test. Dashed lines represent soil data: gray, bulk soil; green and brown, Ca(NO_3_)_2_ extractable Cd (2 * standard deviation of the mean of *n* = 2 experimental replicates). Data from the flowering growth stage was published previously (Wiggenhauser et al., 2021).

**FIGURE 2:**
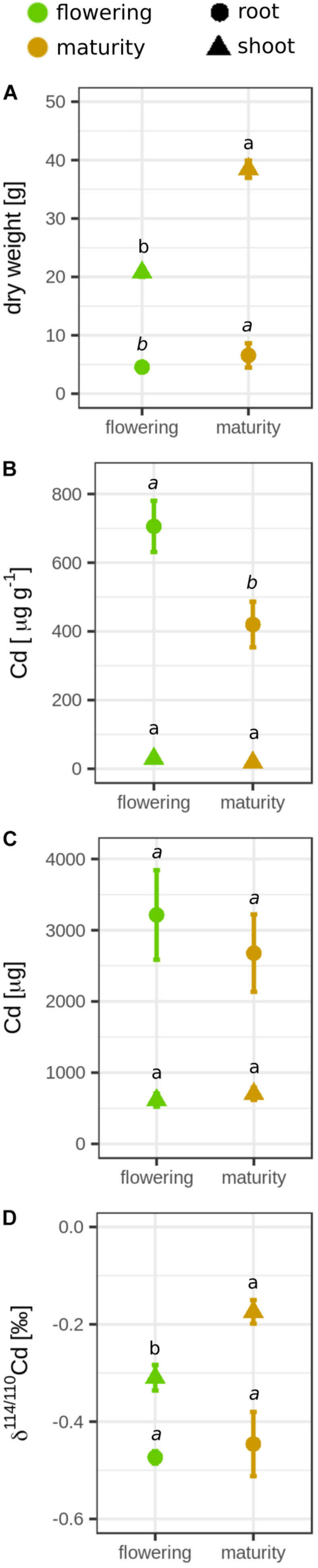
Mean **(A)** dry weight (DW), **(B)** cadmium (Cd) concentration, **(C)** Cd mass, and **(D)** Cd isotope ratio of the roots and shoots of rice plants that were harvested at flowering and maturity. Error bars represent the standard deviation of *n* = 4 experimental replicates. Letters denote significant differences of the mean (regular = shoot, italic = root) that were determined by either a [**B,C** (root), **D**] Welch’s *t-*test or [**A,C** (shoot)] Wilcoxon rank sum test. Data from the flowering growth stage were published previously (Wiggenhauser et al., 2021).

During grain filling, the DW of the entire rice plant increased by factor 1.8 (flowering, 25.3 ± 0.66 g; maturity, 45.9 ± 3.22 g; Figure 1A and Supplementary Table 2), while the Cd concentration in the rice decreased by factor 2 during grain filling (flowering, 151 ± 24.9 μg g^–1^; maturity, 75.0 ± 11.0 μg g^–1^; Figure 1B and Supplementary Table 3). These Cd concentrations in the rice plants were 5- to 10-fold higher than the initial Cd concentration in the soil [15 mg (kg soil)^–1^]. In total, 16% of the Cd that was initially present in the soil (22,500 μg) was transferred from the soil to the rice. The Cd mass in the plant remained constant during grain filling (flowering, 3828 ± 709 μg; maturity, 3380 ± 590 μg, Figure 1C). The Cd isotope ratio in the whole plant did not significantly change during grain filling (Δ^114/110^Cd_*maturity–*__*flowering*_ = 0.06 ± 0.05‰, Figure 1D). However, changes were observed between root and shoot during grain filling. The shoot DW increased during grain filling by factor 1.8, while the root DW did not significantly differ in this growth period (Figure 2A). The Cd concentration in the shoot significantly declined from 29.9 ± 4.61 μg g^–1^ to 18.3 ± 2.43 μg g^–1^, while the Cd mass in the shoot did not significantly change during grain filling (Figures 2B,C). In the same period, the shoot became enriched in heavy isotopes (Δ^114/110^Cd_*maturity–*__*flowering*_ = 0.14 ± 0.04‰), while the isotope composition of roots remained constant (Figure 2D).

Soil pH was lower in the vegetative (mean, 4.52 ± 0.67) compared to grain filling period (4.98 ± 0.23, Supplementary Table 4). However, at the time of sampling, the soil pH was slightly higher at flowering (5.52 ± 0.24) than at maturity (5.14 ± 0.11). At these sampling time points, neither soil Eh (flowering, 195 ± 22.3 mV; maturity, 217 ± 5.10) nor the Cd concentration in the Ca(NO_3_)_2_-extractable soil Cd pool differed [flowering, 3.52 ± 2.10 mg (kg soil)^–1^; maturity, 3.65 ± 0.23 mg (kg soil)^–1^]. However, the Ca(NO_3_)_2_-extractable soil pool slightly changed its isotope composition in this period (flowering, δ^114/110^Cd = −0.18 ± 0.04‰; maturity, −0.07 ± 0.04‰).

### Cd in Straw, Nodes, Flag Leaves, Panicle, and Grains at Maturity

**FIGURE 3:**
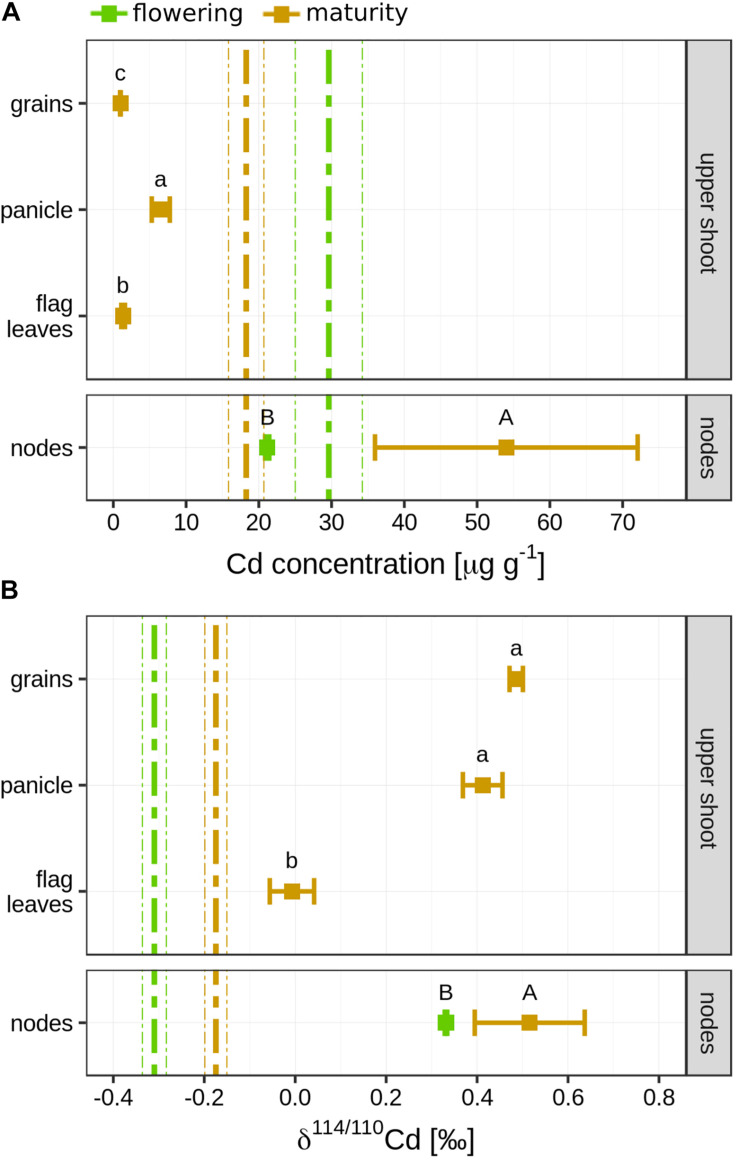
**(A)** Mean Cd concentration in different shoot parts. Upper shoot: error bars denote the standard deviation of *n* = 4 experimental replicates sampled at maturity. Letters indicate statistical difference of the mean determined by using a Kruskal–Wallis test. Dashed vertical line indicate mean Cd concentration (±1 sd) of the entire shoot. Nodes: error bars denote the standard deviation of *n* = 2 experimental replicates at flowering and maturity. **(B)** Mean Cd isotope ratios in different shoot parts. Upper shoot: error bars denote the standard deviation of *n* = 4 experimental replicates sampled at maturity. Letters indicate statistical difference of the mean determined by using a Kruskal–Wallis test. Vertical line indicates mean isotope ratio (±1 sd) of the shoot. Nodes: error bars denote the two standard deviations (±2 sd) of *n* = 2 experimental replicates at flowering and maturity.

The Cd concentration in the nodes (21.2 ± 0.30 μg g^–1^) was slightly lower compared to the shoot Cd concentration at flowering (29.6 ± 4.61 μg g^–1^, Figure 3A and Supplementary Table 3). At maturity, the mean Cd concentration in the nodes (54.0 ± 18.04 μg g^–1^) was three times higher than the Cd concentration in the entire shoots (18.3 ± 2.43 μg g^–1^). This shift in concentration was accompanied by an enrichment of heavy isotopes in the nodes during grain filling (Δ^114/110^Cd_*maturity–*__*flowering*_ = 0.18 ± 0.12‰, Figure 3B). At maturity, Cd concentration in the shoot was significantly higher than in the panicle (6.52 ± 1.24 μg g^–1^), flag leaves (1.35 ± 0.25 μg g^–1^), and grains (0.98 ± 0.05 μg g^–1^, Figure 3A). Only a minor part of the total Cd in the shoot accumulated in these plant parts (Supplementary Table 5). The smallest fraction was found in the flag leaves (0.27 ± 0.63%), followed by grains (0.67 ± 0.25%) and panicle (2.61 ± 0.35%). Compared to the shoot at maturity, the flag leaves (Δ^114/110^Cd_*flag.leaf–*__*shoot*_ = 0.17 ± 0.05‰), panicle (Δ^114/110^Cd_*panicle–*__*shoot*_ = 0.59 ± 0.05‰), and grains (Δ^114/110^Cd_*grains–*__*shoot*_ = 0.66 ± 0.03‰) were strongly enriched in heavy isotopes (Figure 3B). Consequently, the straw was isotopically slightly lighter (δ^114/110^Cd = −0.20 ± 0.02‰) than the shoot (δ^114/110^Cd = −0.17 ± 0.02‰).

### Cd Solid-State Speciation in Soil and Rice

**FIGURE 4:**
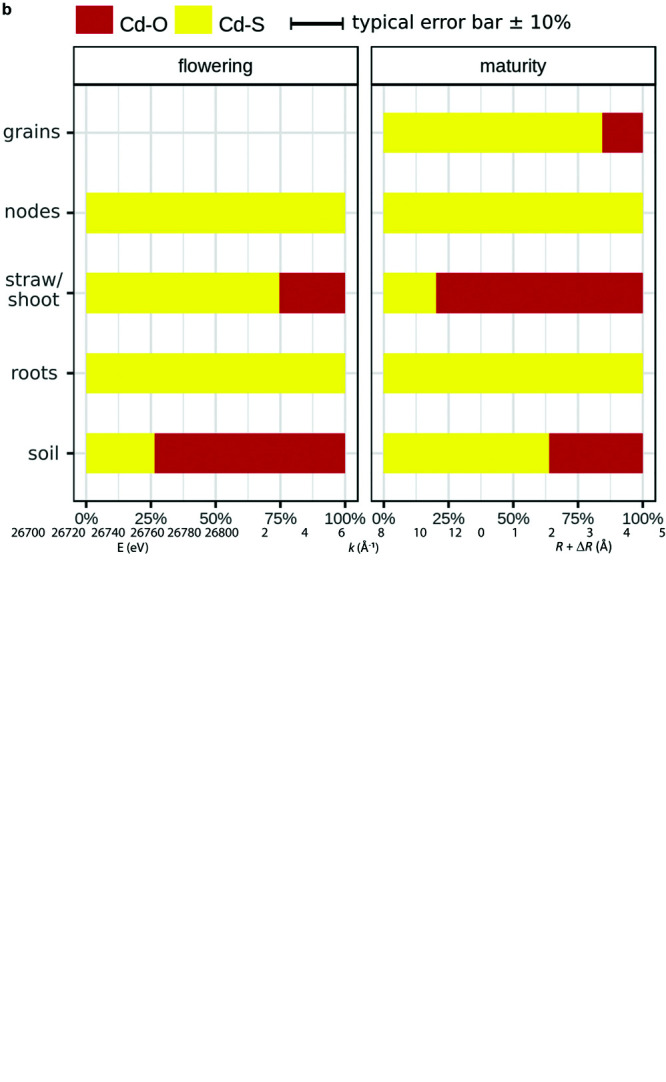
**(a)** K-edge X-ray absorption near edge structure (XANES) **(A)**, extended X-ray absorption fine structure (EXAFS) **(B)**, and Fourier transformed **(C)** spectra for soils and rice parts that were sampled at flowering (Flow) and maturity (Mat) stage, and for selected Cd reference compounds. For some spectra (e.g., grain_Mat_1), only the XANES spectrum was analyzed because of the low Cd concentration in these samples. Dashed line represents linear combination fits, performed on the XANES **(A)** and *k*^2^ EXAFS spectra (**B**, and their FTs in panel **C**). Spectra from soil and root at flowering were taken from in Wiggenhauser et al. (2021). **(b)** Cadmium (Cd) speciation in different compartments of the soil–rice system at different growth stages. “Cd–O” denotes Cd bound to COOH/OH groups of organic matter or Cd sorbed to minerals, and “Cd–S” represents Cd bound to thiols (R-SH). Detailed results of the linear combination fits can be found in Supplementary Table 6. The error bar represents a typical precision for Cd speciation analysis with XAS (see the section “Statistics”). Speciation results from soil and root at flowering were taken from Wiggenhauser et al. (2021).

XAS was used to obtain information about storage forms of Cd in soils and plants. XANES and EXAFS spectra showed contrasted features (Figure 4). For example, for the nodes, the low amplitude of the main peak of the XANES part and the position of the first shell of the Fourier transformed (FT) EXAFS spectrum suggested S ligands for Cd, whereas for the initial soil, spectral features suggested O ligands for Cd. LCF of XANES and EXAFS spectra confirmed the presence of O and S ligands for Cd (Figure 4 and Supplementary Table 6). In the soil at flowering, 27% of the Cd was bound to S groups (Cd–S, corresponding to Cd complexes with thiols), while 73% of the Cd was bound to O groups (Cd–O, corresponding to Cd bound to COOH/OH groups and/or Cd sorbed to minerals). During grain filling, the Cd–S pool increased to 64%, while the remaining Cd was bound to O. In the roots, Cd was entirely bound to S regardless of the growth stage. In contrast, the Cd storage forms changed in the shoots during grain filling. At flowering, Cd–S forms dominated (75%), while at maturity, Cd was mainly bound to O (80%). Cd in the nodes was exclusively bound to S at both growth stages. In the grains, Cd was mainly bound to S (85%).

### Comparison of Cd With Micronutrients

**FIGURE 5:**
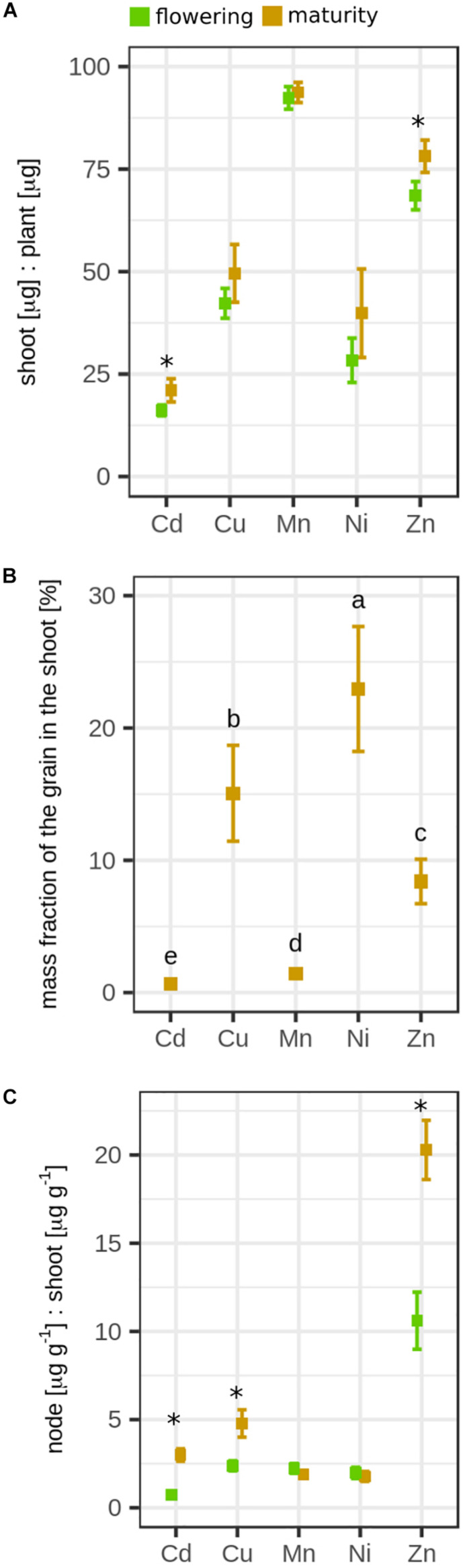
**(A)** Shoot/plant mass ratio of cadmium (Cd) and micronutrients at flowering and maturity. Error bars denote standard deviation of the mean of *n* = 4 experimental replicates for which the mass ratios were calculated individually. Asterisks denote significant difference in the mean between flowering and maturity determined by a Welch’s *t* test. **(B)** Mass fraction of Cd and micronutrients in the grain compared to the shoot. Error bars denote the standard deviation of *n* = 4 experimental replicates. Letters denote significant differences of the mean determined by a Kruskal–Wallis test. **(C)** Enrichment of Cd and micronutrients in the nodes. Values show the concentration ratio of node/shoot at flowering and maturity. Error bars denote the standard deviation of *n* = 4 experimental replicates. Asterisks denote significant difference in the mean between flowering and maturity determined by a Welch’s *t* test.

**FIGURE 6:**
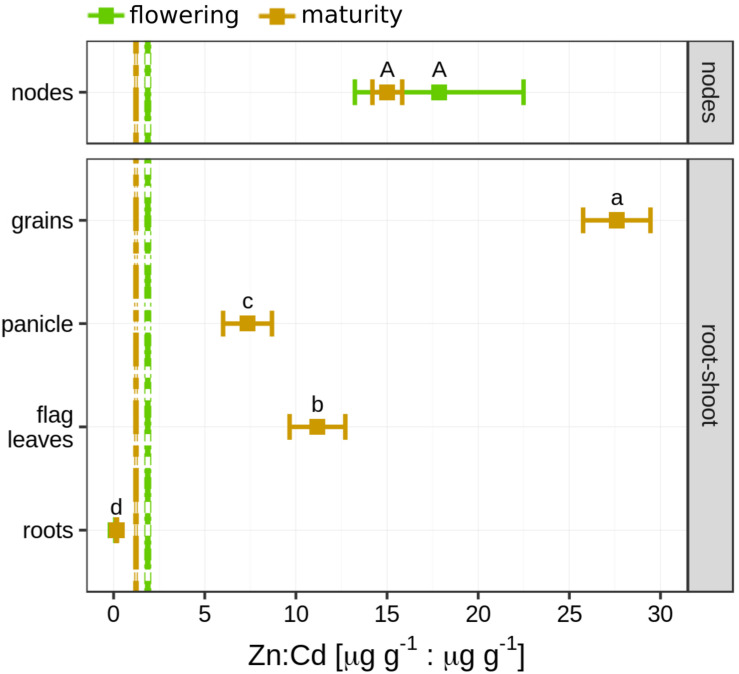
Zn:Cd mass ratios in different plant parts. Error bars denote the standard deviation of *n* = 4 experimental replicates and letters denote statistical difference of the mean determined using ANOVA (non-transformed). Except for nodes: the standard error denotes 2sd of *n* = 2 experimental replicates and statistical difference was evaluated visually. Vertical dashed lines denote Zn:Cd mass ratios in the shoot (flowering) and straw (maturity). Thin lines denote the standard deviation (sd) of *n* = 4 experimental replicates. The Zn:Cd mass ratio of the root at flowering is plotted but not visible since it is identical with the root at maturity.

Unlike Cd, the soil to plant transfer of Mn and Zn increased 1.8-fold during grain filling (Supplementary Table 2). Furthermore, a minor fraction of the total mass of Cd present in the plant was present in the shoot (16–21%) compared to Zn (69–78%) and Mn (92–94%, Figure 5A). During grain filling, the Cd and Cu concentration in the shoot significantly decreased by 38 and 28%, respectively, but remained constant for the other elements (Supplementary Tables 2, 3). The accumulation of micronutrients and Cd in the grains differed (Figure 5B and Supplementary Table 7). The mass fraction of an element in the grain compared to the entire shoot ranged from 0.7 to 22% and was highest for Ni followed by Cu > Zn > Mn > Cd. The Cd, Cu, Mn, and Ni concentration in the nodes was less than five times higher than in the shoot at both growth stages (Figure 5C). For Zn, the concentrations in the nodes were 10 and 20 times higher compared to the shoot at both growth stages. During grain filling, the Zn, Cd, and Cu concentrations in the nodes also increased compared to the concentrations of these elements in the shoot and straw. Among the micronutrients measured, Cd is thought to take similar pathways through plants as Zn (Yamaji and Ma, 2014), which might be related to their chemical similarity (Maret and Moulis, 2013). The Zn/Cd mass ratio was <1 (μg g^–1^/μg g^–1^) in the roots and ranged within 1–2 (μg g^–1^/μg g^–1^) in the shoot and straw (Figure 6). More Zn than Cd accumulated in the panicle, nodes, flag leaves, and grains compared to Cd (7–28 μg g^–1^/μg g^–1^) at both growth stages.

## Discussion

### Root Losses and Sulfur Reduced the Cd Soil-to-Rice Transfer During Grain Filling

The DW of the whole plant increased during grain filling (Figure 1A) including the shoot DW that increased by factor 1.8 (Supplementary Table 2). This increase is high compared to rice plants that grow in upland conditions in the field where the shoot DW slightly increases or decreases during the grain filling period (Fageria, 2014). The unusual increase in shoot DW might be related to the controlled growth conditions in pots including permanent water supply as well as constant light, temperature, and humidity during rice growth (Köhl, 2015).

Although the plant continued to grow during grain filling, no significant Cd transfer from soil to rice was detected in this growth period (Figure 1C). Since the Cd concentration in the root was 23 times higher than in the shoot, small losses of roots could have a strong impact on the Cd mass balance of the entire plant. Root losses during the separation of the roots from the soil and during the root washing procedure may have been larger at maturity compared to flowering, since the roots may have already started to decompose. Alternatively, membrane proteins that are important for root uptake from soil to plant for Mn and Zn have been reported to also take up Cd (Nakanishi et al., 2006; Lee and An, 2009; Sasaki et al., 2012). The plant uptake of Zn and Mn increased by factor 1.8 during grain filling, which is equal to the increase in dry weight during grain filling (Supplementary Table 2). These results suggest that membrane transporters that were potentially utilized by Cd were still active during grain filling. Together, rather root losses at harvest than a downregulation of root membrane transporters reduced the soil-to-plant transfer during grain filling.

In addition to root losses, the Cd plant availability might have been changed during grain filling. The Cd in soil was predominately bound to O at flowering and to S at maturity (Figure 4). Since the plants grew in upland conditions in aerated conditions, the coordination of Cd to O groups can be ascribed to Fe and Mn oxides, clay minerals, and soil organic matter (SOM) (Loganathan et al., 2012). A major fraction of S is present in aerated agricultural soils as organic sulfur (Wilhelm Scherer, 2009). Within the organic S soil pool, a significant fraction of S can be thiols (R-SH, Turner et al., 2016). Cd and Cu have a higher affinity to thiols compared to Zn or Mn (Sóvágó and Várnagy, 2013; Essington, 2015). Furthermore, Cd binds more strongly to thiols than carboxy groups of soil organic matter (Karlsson et al., 2007). Similar to Cd, the transfer of Cu from soil to rice was negligible during grain filling (Supplementary Table 2). Hence, it is likely that the shift from mainly Cd–O to Cd–S groups during grain filling largely contributed to the immobilization of Cd in soils. During grain filling, we observed that the soils in the pots became compacted, and the water consumption of the rice declined. Thus, the soil may have been less aerated during grain filling; this may have increased the abundance of reduced S groups in the soil (Zhao et al., 2006; Prietzel et al., 2007). This view is partly supported by the Ca(NO_3_)_2_-extractable Cd soil pool (Figure 1D) that mimics plant available Cd (Gray et al., 1999). During grain filling, this Cd soil pool became enriched in heavy isotopes (Figure 1D). In the acidic soil conditions of this study, this extract may well represent the plant available Cd pool, since the major fraction of Cd in the soil solution is present as Cd^2+^ as previously shown in Wiggenhauser et al. (2021). The increased binding of Cd to S in the soil and the isotope shift in the Ca(NO_3_)_2_-extractable Cd soil pool during grain filling could correspond to Cd binding to thiol groups of SOM that favorably bind light isotopes compared to O and N groups (Yang et al., 2015; Zhao et al., 2021). However, we observed no clear change in the Cd concentration in the Ca(NO_3_)_2_-extractable Cd soil pool (Supplementary Table 4). The absence of a change in Cd concentration in the soil extract during grain filling might be related to the large variation in Cd concentration that was found in the extract at flowering. Typically, the Cd concentration varies stronger than Cd isotope ratios in experimental replicates of extracts or plants, as shown in this study (Supplementary Table 3) and elsewhere (Wei et al., 2016, 2018; Wiggenhauser et al., 2016). Hence, the Cd isotopes may be more robust than Cd concentrations to temporal changes in a soil extract taken from fresh soils. Altogether, our results suggest that the increased binding of Cd to S in the soil contributed to a decrease in the plant availability of Cd in the soil and, therefore, the transfer of Cd from soil to plant.

### Transport of Cd Between Root and Shoot During Grain Filling

More than three quarters of the Cd in the plant was allocated in the roots (Figure 2C) where Cd was fully bound to S (Figure 4b). Previous studies showed that large fractions of Cd in the roots participate in the operationally defined apoplastic Cd pool (>60–80%; Redjala et al., 2009; Liu et al., 2014). This Cd pool is thought to be mostly bound to carboxylic groups of pectins, which would correspond to Cd–O. Our result that 100% of the Cd in the root was bound to S suggests that only a small fraction of Cd was bound to the root cell walls of our study. These XANES results are supported by root extractions of a previous study that revealed that a minor fraction of Cd in the root was extractable using calcium chloride (<1.5%, Wiggenhauser et al., 2021). The different results observed in our study and the abovementioned studies could be related to distinct growth conditions (hydroponic, Redjala et al., 2009) and the distinct soil pH in Liu et al. (2014). The negatively charged root apoplast is a major sorption side in hydroponic systems, and in alkaline soil conditions, sorption of Cd to pectins is likely to be higher than in acidic conditions (Loganathan et al., 2012).

Cd was strongly retained in the roots that where enriched in light isotopes compared to the shoots (Figure 2D). The same pattern was also observed in wheat, barley, and cacao seedlings (Wiggenhauser et al., 2016; Imseng et al., 2019; Moore et al., 2020). For hydroponically grown rice seedlings that were exposed for 3 days to 0.1 μM Cd, the isotope ratios between root and leaves were isotopically not distinguishable in wild-type plants, but a slight enrichment of heavy isotopes was observed in leaves when the root vacuolar transporter OsHMA3 was overexpressed (Zhang et al., 2021). Our Cd speciation and isotope data strongly suggest that Cd in the roots was bound to chelating thiols such as phytochelatin (Nocito et al., 2011; Wiggenhauser et al., 2021) after it was transported into the vacuole by OsHMA3 (Shao et al., 2017). The membrane transport and/or the chelation of Cd to thiols leads to the retention of light isotopes in the roots (Wiggenhauser et al., 2021). Thiol complexes with heavy isotopes are less stable at equilibrium, which leads to enrichment of heavy isotopes in the Cd^2+^ species that can be transported out of the vacuole and then loaded into the xylem (Wiggenhauser et al., 2021; Zhao et al., 2021). Exporting of Cd out of the vacuole and loading into the xylem requires membrane transport proteins that could further fractionate Cd isotopes (Wiggenhauser et al., 2021). The processes that were involved into the retention of Cd in roots seemed to be very selective, since all micronutrients measured in this study were less strongly retained in the roots compared to Cd (Figure 5A). During grain filling, Cd was strongly retained in the roots as indicated by the Cd concentration, Cd speciation, and Cd isotope ratios in the roots that remained unchanged during grain filling. Our data strongly suggest that chelating thiols retained Cd in the roots until harvest despite the onset of plant senescence during grain filling.

In contrast to the roots, the Cd concentration and the Cd isotope ratios in shoots changed during grain filling (Figures 2B,D). The shoot Cd concentration decreased likely due to a dilution effect since the DW growth rate was larger than the Cd root-to-shoot transfer rate (Figures 2A,B). The Cd isotope ratio in the shoot increased through root-to-shoot transport of heavy Cd isotopes and/or shoot-to-root transfer of light Cd isotopes during grain filling. Both transport pathways have been previously reported for cereals (Cakmak et al., 2000; Riesen and Feller, 2005; Kashiwagi et al., 2009; Yan et al., 2019). For the root-to-shoot transport, a small mass of Cd that is strongly enriched in heavy isotopes would be needed to enrich the shoot in heavy isotopes. This would imply a stronger retention of light isotopes through Cd–S chelation during the grain filling compared to the vegetative growth period. This strong retention would lead to a more pronounced enrichment of heavy isotopes in the Cd^2+^ species that can be transported from roots to shoots (Wiggenhauser et al., 2021). In contrast, the shoot-to-root transport would imply a mobilization of light isotopes from the shoots. However, other phloem sinks than the roots such as the grains (Tanaka et al., 2007) were strongly enriched in heavy isotopes (Figure 3B), making it unlikely that light isotopes were transported from shoot-to-root during grain filling. Therefore, our data suggest that Cd root-shoot exchange was very limited, and only a small fraction of Cd in the roots was transported to the shoots during grain filling.

Unlike in the roots, Cd in the shoots was not solely stored as Cd–S but also as Cd–O (Figure 4). Generally, less is known about the function of storage forms in the shoot compared to the root (Sterckeman and Thomine, 2020). Our observation that large fractions of Cd in the shoot were bound to O donor atoms coincides with previous studies that detected larger fractions of Cd bound to Cd–O in shoots of rice compared to roots (Yamaoka et al., 2010; Yan et al., 2016). In addition, most measurements of Cd storage forms in plants have been conducted in Cd accumulator plants. In these plants, Cd–O is the major storage form in shoots (Küpper et al., 2004; Ueno et al., 2005; Tian et al., 2011; Huguet et al., 2012; Isaure et al., 2015). The Cd–O complexes may correspond to organic acids that complex Cd in vacuoles to attenuate Cd efflux from the vacuole (Ueno et al., 2005). Cd–organic acids complexes are most stable in acidic compartments (e.g., vacuole, xylem, apoplast) but are generally less stable than Cd–thiol complexes (Filella et al., 1999; McLaughlin et al., 1999; Sóvágó and Várnagy, 2013). Cd–O storage forms have been also interpreted as Cd in the apoplast that binds to cell wall components (Isaure et al., 2015). Extraction-based analysis of Cd compartmentalization in rice shoots revealed that large fractions of Cd in the shoot (>40%) are located in the apoplast (Yu et al., 2012; Siebers et al., 2013; Zhang et al., 2014). This Cd accumulation in the apoplast may derive from inefficient loading of Cd from the xylem into the symplast in the shoot (Olsen and Palmgren, 2014) that requires membrane influx transporters that are not identified yet (Wang W. et al., 2019). Furthermore, the small fraction of Cd–S forms in the shoots of Cd accumulator plants was ascribed to Cd detoxification by thiols (Isaure et al., 2015). In summary, the Cd–O storage forms in rice shots may either represent Cd–organic acid complexes in vacuoles and/or Cd bound to cell walls in the apoplast.

The change in the Cd storage from Cd–S to Cd–O during grain filling (Figure 4b) was likely related to senescence of stems and leaves. In this period, catabolic activities recycle nutrients such as N, S, and micronutrients and transport them towards the grain (Pottier et al., 2014; Maillard et al., 2015; Etienne et al., 2018). This recycling step includes an organized degradation of organelles and organic compounds, including proteins. Significant fractions of Cd can be present in organelles in rice shoots (5–17%, Yu et al., 2012; Siebers et al., 2013; Zhang et al., 2014) and/or replacing nutrients such as Zn in proteins in plants (Tang et al., 2014). Hence, in our study, the Cd that was bound to S in the shoots at flowering could have been remobilized toward the grains during grain filling (Kashiwagi et al., 2009; Wang et al., 2018). On average, 313 μg of Cd dissociated from S during grain filling. The quantity of Cd that dissociated from S was more than 10 times larger than the Cd mass that accumulated in the upper shoot (<25 μg) or grains (<5 μg, Supplementary Tables 3, 5). In addition, a transfer of the Cd that was bound to S from straw to grain would have led to an accumulation of light isotopes in the grains or upper shoots; this was not the case (Figure 3A). A shoot-to-root transfer of Cd that was bound to S can also be excluded. Thus, we propose that enzymatically controlled processes during leave senescence may have dissociated Cd from S, and the majority of Cd then bound to O donors of organic acids or cell walls that contributed to the immobilization of Cd in straw.

### Transport of Cd From Straw to Grain

**FIGURE 7:**
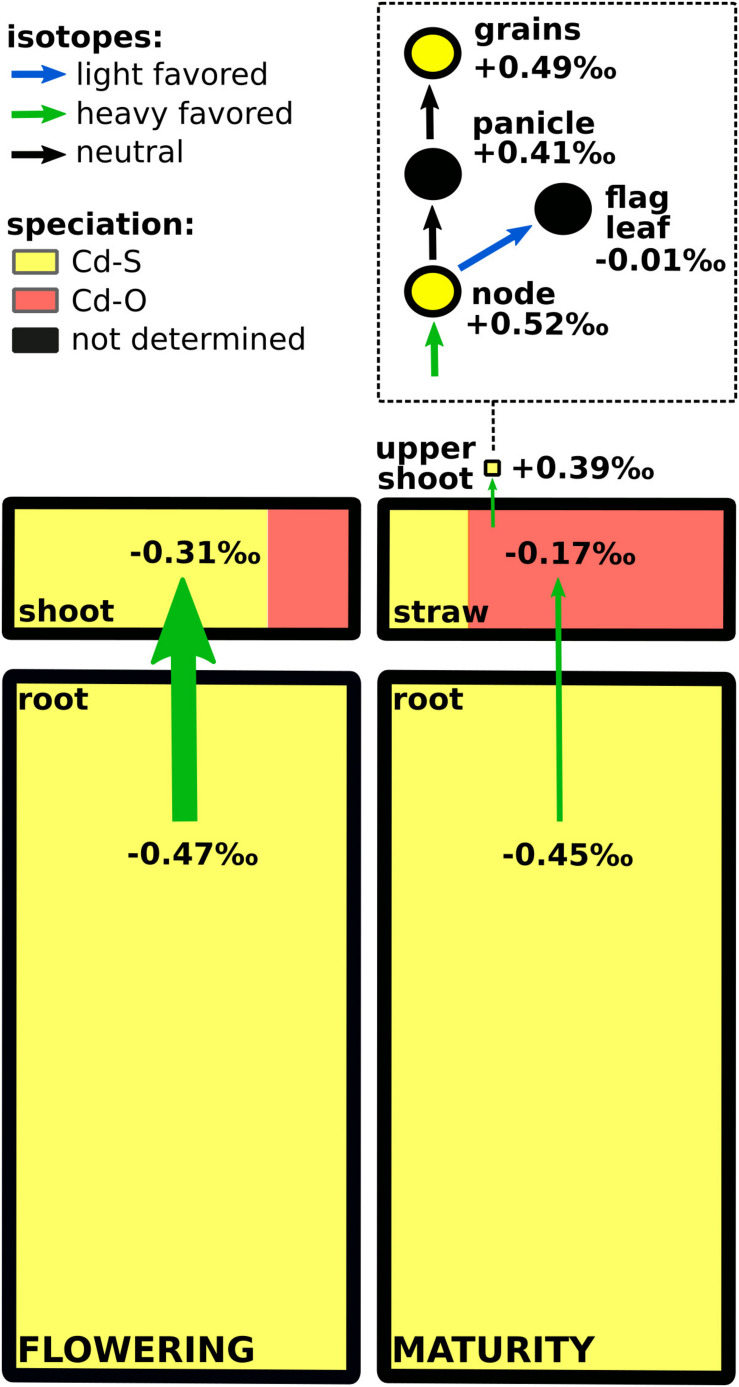
Summary of Cd isotope fractionation and Cd storage forms in rice that grew on a Cd contaminated soil in upland conditions. Boxes (flowering left and maturity right) are proportional to the mass of Cd in each compartment (root, shoot, straw, and upper shoot), while circles are not proportional to the Cd mass (node, flag leaf, panicle, and grain). Isotope values are given as δ^114/110^ Cd. Cd-O denotes Cd bound to COOH/OH groups and Cd-S represents Cd bound to thiols (R-SH). In the roots, Cd was fully bound to S despite plant senescence during the grain filling period. In the shoots, Cd was bound to S and O and changed during grain filling. Both, Cd-O and Cd-S storage forms may contribute to the retention of Cd in roots and shoots since only a minor fraction of Cd accumulated in the upper shoot and ultimately in the grains. The grains were strongly enriched in heavy isotopes, which is likely induced through strong chelation of Cd by thiols and by membrane transport. Isotope fractionation and Cd speciation in the upper shoot suggest that heavy Cd isotopes are favorably transported from xylem to phloem and Cd-S may persist during the transport from nodes via panicle into the grains.

Previous studies evidenced that a significant fraction of the Cd in the grain can be remobilized from the stems and leaves into the grains of rice and wheat (Rodda et al., 2011; Wang et al., 2018; Yan et al., 2018, 2019). In our study, the Cd mass in the grain was more than 100 times smaller than the Cd mass in the straw and also when compared to micronutrients (Figures 5B, 7). Therefore, Cd was strongly retained in the shoot and less mobile than the micronutrients within the shoot during grain filling. The grains were strongly enriched in heavy isotopes compared to the straw or the roots (Δ^114/110^Cd_*grains–*__*straw*_ = 0.69 ± 0.02‰, Δ^114/110^Cd_*grains–*__*roots*_ = 0.93 ± 0.07‰, Figures 2, 3). Hence, the Cd isotope fractionation within the plant can strongly contribute to the enrichment of heavy Cd isotopes in the grains. This enrichment of heavy isotopes in rice grains corresponds with previously reported Cd isotope ratios for rice grown on contaminated soils in the field (Zhang et al., 2021) as well as for wheat and barley that grew on non- or moderately contaminated soils (Wiggenhauser et al., 2016; Imseng et al., 2019). In addition to isotope ratios, we measured the Cd speciation in the shoot and straw, which may partly control the shoot-to-grain transfer of Cd. Since the Cd–O storage forms in the rice straw are predicted to be enriched in heavy isotopes compared to the Cd–S storage forms (Yang et al., 2015; Zhao et al., 2021), a small fraction of Cd that was stored as Cd–O in the shoot could have been a source of Cd in the grain.

In rice, the nodes play a crucial role in distributing micronutrients and Cd within the shoot (Fujimaki et al., 2010). Nodes contain xylem and phloem vessels as well as parenchyma cells that connect these long-distance transport vessels (Yamaji and Ma, 2014). Membrane transporters regulate xylem and phloem loading and unloading of Cd and nutrients. This includes the transfer of Cd from the xylem to the phloem that is directed to non- or less transpiring organs such as the grains or young plant parts (Herren, 1997; Tanaka et al., 2007). The node analyzed in this study (node I) connects the upper shoot (flag leaf with panicle and grains) with the lower shoot (Yamaji and Ma, 2014). A major pathway for Cd (and Zn) to reach this node is the transfer of Cd from node to node, which requires a xylem to phloem transfer in the nodes (Fujimaki et al., 2010; Yamaji and Ma, 2014). This pathway is characterized by elevated concentrations of Cd and Zn in the nodes as also found in our study (Figure 5C). At maturity, the Cd concentration in node I was 2.1 and 18 times higher compared to the straw and the upper shoot, respectively (Supplementary Tables 3, 5). In addition, the nodes were strongly enriched in heavy isotopes compared to the lower shoots and also heavier than the flag leaves (Figure 3B). Hence, the Cd transfer from the xylem to the phloem leads to an enrichment of heavy isotopes in node I. Furthermore, the flag leaves were enriched in heavy isotopes compared to the lower shoot (Figure 3B). This indicates that Cd becomes successively enriched along the transport from node to node where lighter isotopes are directed toward the transpiring leaves and heavy isotopes are loaded into the phloem and transported toward node I and finally into the panicle. The xylem to phloem transfer in the nodes comprises several steps that could fractionate Cd isotopes. To date, it is known that Cd in the acidic xylem is mostly present as Cd^2+^ (Yoneyama et al., 2015), xylem unloading of Cd into companion cells that connect the xylem and phloem requires membrane transport (Satoh-Nagasawa et al., 2012; Yamaji and Ma, 2014), and the predominant Cd species in the nodes is Cd–S (Figure 4b) as also reported in Yamaguchi et al. (2012). The similar Cd speciation between the node and grain (Figure 4b) and the absence of Cd isotope fractionation between the node, panicle, and grains (Figure 3B) suggest that rather membrane transport during xylem unloading of Cd^2+^ than Cd speciation governed the isotope fractionation during xylem to phloem transfer in the nodes.

The results that Cd was fully bound to S in the nodes, the majority of Cd in the grains was bound to S (Figure 4b), and no isotope fractionation occurred between the node, panicle, and grains (Figure 3B) suggest that Cd was transported as Cd–S from the nodes into the grains. A transport from the node to the grain without ligand exchange would require that Cd is transported as Cd–S complex from the node via phloem directly into the grain crease (Yan et al., 2020). Little is known how Cd and metals are transported from the nodes into the phloem. For Zn, rice mutants with knocked out genes that encode metallothionein in node I decreased the Zn transport from the node to the grain (Lei et al., 2020). These proteins with S donor ligands seem to bind Zn also in the companion cells that are directly connected to the phloem without a membrane that separates these cells from the phloem. If Cd follows the pathway of Zn from the node to the grain, a transport of Cd–S without isotope fractionation between the node and the grain could be possible. However, the transfer of Zn from the node to the grain was more efficient than for Cd as shown by the high Zn/Cd ratio in the grains (Figure 6). In addition, Yamaguchi et al. (2012) showed, by using μXANES, that the location of Zn and Cd in the nodes can differ. These results suggest that the pathways of Cd and Zn from the node to the grain may not be identical. Finally, Cd speciation measurements in the phloem sap of rice (Kato et al., 2010) and other plant species (Mendoza-Cózatl et al., 2008; Hazama et al., 2015) revealed that thiol containing ligands may bind Cd in the phloem. Together, the few findings of Cd speciation in nodes and phloem sap as well as first isotope data suggest that Cd may be transported from the node to the grain in a complexed Cd–S form (Figure 7). However, more studies are needed to understand the role of xylem to phloem transport and the transport from the node to the grain on Cd isotope fractionation.

### Sulfur a Major Binding Site in Rice Grains

In the grains, almost all Cd was bound to S (Figure 4b). This observation coincides with previously published size exclusion ICP-MS data that identified S-bearing proteins as the major storage form of Cd in rice (Wei et al., 2017). In addition, μXANES studies revealed that large fractions of up to 92% of Cd can be bound to S in rice and wheat grains (Gu et al., 2020; Yan et al., 2020). The consistent findings that S can be an important binding site for Cd may have implications on Cd absorption in human nutrition (Gu et al., 2020). Micronutrients such as Zn and iron (Fe) can be strongly bound to phytate in cereals, which inhibits the absorption of minerals (Persson et al., 2009, 2016; De Brier et al., 2016; Grases et al., 2017) and can potentially also reduce the absorption of Cd (Schlemmer et al., 2009). Since S is a major binding for Cd in cereals, Cd binding to phytate may play a subordinate role compared to Zn and Fe. In rice, the bioaccessibility of Cd can vary from 17 to 84 (Li et al., 2019). This variation can result from the dietary content of, e.g., Zn and Fe (Reeves and Chaney, 2008) and how the rice meals are cooked (Zhuang et al., 2016). To date, the role of Cd speciation, especially binding of Cd- to S-bearing ligands and proteins on the bioavailability, is not known yet.

## Implications and Outlook

We combined for the first time Cd mass balances, Cd isotopes, and Cd speciation analyses to elucidate the role of Cd storage forms during Cd remobilization from the root and shoot into the grain (Figure 7). We highlighted that Cd was strongly retained in roots and shoots and, overall, less mobile than micronutrients in aerated soil–rice systems. In the roots, Cd–S was the sole storage form, and it did not change during grain filling. In shoots, Cd was stored as Cd–S and Cd–O, and the proportion of Cd–O increased during grain filling. Our data strongly suggest that this change in the storage form did not lead to significant mobilization of Cd. Tissues that served as phloem sinks (grains) or that interlink Cd sources with phloem sinks (nodes) were strongly enriched in heavy isotopes. Together, these main findings showed that Cd can be locked in roots as Cd–S despite that plant senescence and Cd immobilization and detoxification differ in roots and shoots. In the latter, the Cd–O storage form also contributes to the immobilization of Cd in shoots. Whether Cd–O refers to organic acids or apoplastic storage with binding to cell walls and how these processes separate Cd from essential micronutrients need to be clarified. In addition, bulk XANES analysis of rice grains showed that Cd–S can be the major Cd storage forms in grains. This shows that Cd binding to the antinutrient phytate may be less important than for Zn or Fe. Future studies should investigate the role of Cd speciation in cereal grain on the Cd bioavailability for humans.

This study highlighted the importance of Cd storage forms on Cd dynamics in soil–rice systems. The findings of this study were generated in a soil–rice system that was characterized by a high Cd and S soil content (Wiggenhauser et al., 2021). Similar studies with soils that differ in Cd and S concentrations may provide further insights to the role of Cd storage forms on Cd dynamics in plants (Cao et al., 2018). Due to Cd accumulation in our bodies, Cd can be harmful for us at concentrations plants can still cope with; the mechanistic understanding of the process that controls Cd in such moderately contaminated systems is highly relevant. Hence, investigating the role of storage forms on Cd dynamics during grain filling in plants in which the Cd concentration is lower than 0.2 mg kg^–1^ would be a next crucial step. However, the sample throughput for Cd isotope analyses significantly decreases at low Cd concentrations due to more time-consuming sample preparation (Wiggenhauser et al., 2016; Barraza et al., 2019). Recent advances for isotope analysis of other elements than Cd demonstrated that it is possible to partly overcome this “bottle neck” for sample throughput (Mahan et al., 2020). To determine Cd storage form, we approached the detection limits of XAS (∼1 mg kg^–1^). However, this detection limit may further improve in the future since the synchrotron facilities are steadily increasing the quality and brilliance of their X-rays.

In rice, Cd is thought to take similar pathways from the root and shoot to the grains as Zn (Yamaji and Ma, 2014). However, Zn seemed to be much more mobile within the rice shoots than Cd. The increased mobility of Zn over Cd in the shoot is either related to selective membrane transport in the shoot or to the storage forms of both elements. In contrast to Cd, light Zn isotopes accumulated in rice and wheat (Arnold et al., 2015; Wiggenhauser et al., 2018), which in indicates that combined speciation and isotope analyses could provide novel insights into the processes that separate Cd from Zn. Currently, the interplay of Cd storage forms/Cd speciation and membrane transporters are not well understood. Binding of Cd to organic ligands may impact the transfer of metals through membranes (Zhao et al., 2016). In a next step, Cd storage forms could be coupled with membrane transporters by comparing wild-type and mutant plants that lack or overexpress specific membrane transporters (Cadiou et al., 2017; Pokharel et al., 2018; Lei et al., 2020; Moore et al., 2020; Wiggenhauser et al., 2021; Zhang et al., 2021).

## Data Availability Statement

The original contributions presented in the study are included in the article/Supplementary Material, further inquiries can be directed to the corresponding authors.

## Author Contributions

MW contributed to the conceptualization, methodology, validation, formal analyses, investigation, data curation, writing-original draft, visualization, project administration, and funding acquisition. A-MA contributed to the conceptualization, methodology, investigation, writing–review, and editing. PT contributed to the methodology and investigation. HB contributed to the writing–review and editing. GS contributed to the conceptualization, methodology, validation, formal analyses, investigation, data curation, visualization, writing–review and editing, and funding acquisition. All authors contributed to the article and approved the submitted version.

## Conflict of Interest

The authors declare that the research was conducted in the absence of any commercial or financial relationships that could be construed as a potential conflict of interest.
