# Supplementary material for: Changes of Cadmium Storage Forms and Isotope Ratios in Rice During Grain Filling
Source: Front Plant Sci. 2021 Apr 29;12:645150. doi: 10.3389/fpls.2021.645150 (PMC8116553; doi:10.3389/fpls.2021.645150)
Supplement: Supplementary file 1 [file Data_Sheet_1.PDF]

Supplementary Material

Changes of cadmium storage forms and isotope ratios in rice during grain filling

Matthias Wiggenhauser[1,2*], Anne-Marie Aucour[3], Philippe Telouk[3], Hester Blommaert[2], Géraldine Sarret[2*]

[1]Institute of Agricultural Sciences, Department of Environmental Systems Science, ETH, Zurich, Switzerland.

[2]ISTerre, Univ. Grenoble Alpes, Univ. Savoie Mont Blanc, CNRS, IRD, IFSTTAR, Grenoble, France

[3]Laboratoire de Geologie de Lyon, Ecole Normale Supérieure de Lyon, Université Lyon 1, Université de Lyon, CNRS, Lyon, France

Correspondence:
matthias.wiggenhauser@usys.ethz.ch
geraldine.sarret@univ-grenoble-alpes.fr

Tables

Table_S 1: Quality assessment....2
Table_S 2: Dry weight and micro nutrients root and shoot....2
Table_S 3: Cadmium concentration, mass balance, Cd isotopes....3
Table_S 4: Soil chemical parameters at flowering and maturity....3
Table_S 5: Dry weight, Cd concentration, Cd mass, Cd isotopes in the upper shoot at maturity....4
Table_S 6: Results of linear combination fits of XANES and EXAFS spectra for the soils and plant parts....5
Table_S 7: Dry weight and micronutrient concentrations in the upper shoot at maturity....6

Figures

Figure_S 1: Overview rice parts analyzed....6

1 Tables

Table_S 1: Quality assessment

sample type	description	element	reference concentration [mg (kg dry weight)$^{-1}$] mean	reference concentration ±sd[d]	measured concentration[a] [mg (kg dry weight)$^{-1}$] mean	measured concentration ±sd[d]	n[d]	recovery digestion[b] %[b] mean
rice shoot[h]	internal ISTerre rice standard		–	–	24.6	1.19	9	–
white cabbage	BCR-679	Cd[a]	1.66	0.07	1.77	0.05	3	107
soil	NIST SRM 2711		41.7	–	38.2	0.32	2	92
rice shoot[h]	internal ISTerre rice standard		–	–	35.9	1.84	9	–
white cabbage	BCR-679	Zn	79.70		92.36	2.06	3	116
soil	NIST SRM 2711		350		345	6.00	2	98
rice shoot[h]	internal ISTerre rice standard		–	–	6.48	0.46	8	–
white cabbage	BCR-679	Cu	2.89		2.93	0.20	3	101
soil	NIST SRM 2711		114		104	2.01	2	91
rice shoot[h]	internal ISTerre rice standard		–	–	1.41	0.06	8	–
white cabbage	BCR-679	Ni	27.0		30.34	1.73	3	113
soil	NIST SRM 2711		20.6		18.2	0.23	2	88
rice shoot[h]	internal ISTerre rice standard		–	–	232	9.05	8	–
white cabbage	BCR-679	Mn	13.3		15.52	0.34	3	117
soil	NIST SRM 2711		638		659	14.7	2	103

[a]*data from Wiggenhauser et al. 2020*
[b]*equal to [measured concentration] / [reference concentration] *100*
[c]*number of processing replicates (individually digested, purified for isotope analysis, and measured)*
[d]*sd = standard deviation of the mean*

Table_S 2: Dry weight and micro nutrients root and shoot

compartment	period/growth stage	DW dry weight (g) mean	DW ±sd[a]		Zn concentration (µg g^{-1}) mean	Zn concentration ±sd[a]		Zn mass (µg) mean	Zn mass ±sd[a]	
whole plant	flowering	25.3	0.66	B	43.5	6.97	A	1101	186	A
whole plant	maturity	45.0	3.22	A	44.1	2.89	A	1979	88.4	A
whole plant	grain filling[b]	*19.69*	*3.29*		*0.62*	*7.55*		*878*	*206*	
root	flowering	4.55	0.70	*b*	76.1	9.31	*a*	351	92.5	*a*
root	maturity	6.55	2.08	*a*	67.8	7.92	*a*	433	81.8	*a*
root	grain filling[b]	*2.00*	*2.2*		*-8.26*	*12.2*		*81.7*	*123*	
shoot	flowering	20.8	0.74	b	36.2	5.26	a	750	97.0	b
shoot	maturity	38.4	1.48	a	40.3	3.42	a	1546	106	a
shoot	grain filling[b]	*17.7*	*1.66*		*4.04*	*6.27*		*796*	*143*	
nodes	flowering	NA	NA		378	103		NA	NA	
nodes	maturity	NA	NA		813	315		NA	NA	
nodes	grain filling[b]	NA	NA		*435*	*166*		NA	NA	
grain	harvest	*4.83*	*1.01*		26.9	1.24		130	25.6	

compartment	period/growth stage	Mn concentration (µg g^{-1}) mean	Mn concentration ±sd[a]		Mn mass (µg) mean	Mn mass ±sd[a]	
whole plant	flowering	209	21.1	A	5284	620	B
whole plant	maturity	206	11.2	A	9291	1158	A
whole plant	grain filling[b]	*-2.56*	*23.9*		*4007*	*1314*	
root	flowering	87.5	25.2	*a*	407	165	*a*
root	maturity	88.8	18.4	*a*	606	328	*a*
root	grain filling[b]	*1.33*	*31.2*		*199*	*367*	
shoot	flowering	235	27.3	a	4876	549	b
shoot	maturity	226	16.1	a	8685	836	a
shoot	grain filling[b]	*-9.46*	*31.7*		*3809*	*1000*	
nodes	flowering	519	26.8		NA	NA	
nodes	maturity	428	37.3		NA	NA	
nodes	grain filling[b]	*-91*	*23*		NA	NA	
grain	harvest	26.1	5.3		125	29.9	

compartment	period/growth stage	Ni concentration (µg g^{-1}) mean	Ni concentration ±sd[a]		Ni mass (µg) mean	Ni mass ±sd[a]	
whole plant	flowering	2.57	0.81	A	64.9	20.2	A
whole plant	maturity	1.90	0.20	A	85.7	12.8	A
whole plant	grain filling[b]	*-0.67*	*0.8*		*20.7*	*23.9*	
root	flowering	10.1	2.61	*a*	47.3	18.1	*a*
root	maturity	8.16	2.21	*a*	52.2	15.1	*a*
root	grain filling[b]	*-1.99*	*3.42*		*4.85*	*23.6*	
shoot	flowering	0.85	0.13	a	17.6	2.27	b
shoot	maturity	0.87	0.15	a	33.5	6.64	a
shoot	grain filling[b]	*0.02*	*0.20*		*15.9*	*7.02*	
nodes	flowering	1.65	0.32		NA	NA	
nodes	maturity	1.51	0.10				
nodes	grain filling[b]	*-0.14*	*0.17*		NA	NA	
grain	harvest	1.60	0.35		7.75	2.35	

compartment	period/growth stage	Cu concentration (µg g^{-1}) mean	Cu concentration ±sd[a]		Cu mass (µg) mean	Cu mass ±sd[a]	
whole plant	flowering	10.9	1.85	A	277	50.2	A
whole plant	maturity	6.93	0.64	B	311	22.0	A
whole plant	grain filling[b]	*-4.01*	*2.0*		*33.6*	*54.9*	
root	flowering	35.1	3.43	*a*	161	38.1	*a*
root	maturity	24.8	3.89	*b*	157	28.0	*a*
root	grain filling[b]	*-10.36*	*5.19*		*-4.09*	*47.3*	
shoot	flowering	5.59	0.67	a	116	12.9	a
shoot	maturity	4.01	0.59	b	154	20.0	a
shoot	grain filling[b]	*-1.58*	*0.89*		*37.7*	*23.7*	
nodes	flowering	13.17	2.74		NA	NA	
nodes	maturity	18.81	0.90		NA	NA	
nodes	grain filling[b]	*5.64*	*1.44*		NA	NA	
grain	harvest	4.78	0.69		23.01	5.70	

[a]*sd = standard deviation of the mean of n = 4 experimental replicates, except for the nodes (pooled to n = 2 replicates (error bar = 2sd) to obtain sufficient material for all analysis). Letters indicate significant differences of the mean (upper case = whole plant,lower case italic = root, lower case regular = shoot) determined Welch›s t-test or Wilcoxon rank sum test. e calculation as desscribed in equation 4-5.*

Table_S 3: Cadmium concentration, mass balance, Cd isotopes

compartment	period/growth stage	Cd concentration			Cd mass			Cd isotopes		
		µg g^{-1}			µg[c]			$\delta^{114/110}$Cd [‰]		
		mean	±sd[a]		mean	±sd[a]		mean	±sd[a]	
whole plant	flowering	151	24.9	A	3828	709	A	-0.45	0.01	A
	maturity	75.0	11.0	B	3380	590	A	-0.39	0.05	A
	grain filling[b]	*-75.90*	*27.3*		*-448*	*922*		*0.06*	*0.05*	
root	flowering	706	74.6	*a*	3214	628	*a*	-0.47	0.01	*a*
	maturity	420	66.5	*b*	2679	543	*a*	-0.45	0.07	*a*
	grain filling[b]	*-285*	*99.9*		*-536*	*831*		*0.03*	*0.07*	
shoot	flowering	29.6	4.61	a	614	91.6	a	-0.31	0.03	b
	maturity	18.3	2.43	b	701	84.6	a	-0.17	0.02	a
	grain filling[b]	*-11.3*	*5.21*		*87.5*	*125*		*0.14*	*0.04*	
nodes	flowering	21.2	0.30	NA	NA	NA	NA	0.33	0.00	NA
	maturity	54.0	18.0	NA	NA	NA	NA	0.52	0.12	NA
	grain filling[b]	*32.9*	*9.03*		NA	NA		*0.18*	*0.06*	
grain	harvest	0.98	0.05		4.73	1.03		0.49	0.01	

[a]sd = standard deviation of the mean of n = 4 experimental replicates, except for the nodes (pooled to n = 2 replicates (error bar = 2sd) to obtain sufficient material for all analysis). Letters indicate significant differences of the mean (upper case = whole plant,lower case italic = root, lower case regular = shoot) determined using a Welch's t-test or Wilcoxon rank sum test (whole plant isotope ratios, Cd mass shoot).
[b]balance calculation as desscribed in equations 4-5.
[c]Cd mass is given as µg $(pot)^{-1}$, one pot contains 1.5kg and 22500 µg Cd

Table_S 4: Soil chemical parameters at flowering and maturity

**period/ growth stage**	**rhizon pH[b]**			**rhizon Eh[b]**			**$Ca(NO_3)_2$ soil extractable Cd**					
							[mg (kg $soil)^{-1}$]			$\delta^{114/110}$Cd [‰]		
%	mean	±sd	n	mean	±sd	n	mean	±sd	n	mean	±2sd	n
vegatative[a]	4.52	0.67	10[c]	235	45.5	10[c]						
grain filling	4.98	0.23	2[c]	233	23.2	2[c]						
flowering	5.52	0.24	3	195	22.3	3	3.52	2.10	2	-0.18	0.04	2
maturity	5.14	0.11	4	217	5.1	4	3.65	0.23	2	-0.07	0.04	2

[a]Data obtained in Wiggenhauser et al. (2020)
[b]soil pH determined using rhizon samplers (see methods)
[C]n = sampling time points during the growth period

Table_S 5: Dry weight, Cd concentration, Cd mass, Cd isotopes in the upper shoot at maturity

**compartment**	**dry weight**			**Cd concentration**			**Cd mass**			**Cd isotopes**		
	g			μg g^{-1}			μg[b]			$\delta^{114/110}$Cd [‰]		
	mean	±sd[a]		mean	±sd[a]		mean	±sd[a]		mean	±sd[a]	
shoot	38.4	1.48		18.3	2.43		701	84.6		-0.17	0.02	
straw	25.6	0.96		25.5	3.02		676	84.3		-0.20	0.02	
flag leaves	1.51	1.14	b	1.35	0.25	b	1.88	1.17	c	-0.01	0.05	c
panicle	2.81	0.69	a	6.52	1.24	a	18.3	6.03	a	0.41	0.04	b
grains	4.83	1.01	c	0.98	0.05	c	4.73	1.03	b	0.49	0.01	a
average/sum	9.14	1.68		2.95	3.10		24.9	6.23		0.39	0.03	

[a]*sd = standard deviation of the mean of n = 4 experimental replicates. For the grain filling balance calcuations (italic), the sd was calculated using error propagation as described in equation 4-5. Letters indicate significant differences of the mean determined using ANOVA for Cd mass fraction*

[b]*Cd mass is given as μg (pot)$^{-1}$, one pot contains 1.5kg and 22500 μg Cd*

Table_S 6: Results of linear combination fits of XANES and EXAFS spectra for the soils and plant parts

Sample ID + fit				Percentages of Cd species			Percentages normalized to 100%		
**Compartment**	**Sample name**	**fit in**	**fitting range (in eV or Å-1)**	**Cd-thiol or mixed/ amorph. CdS[a]**	**Cd-O**	**sum**	**Cd-thiol or mixed/ amorph. CdS**	**Cd-O**	**R factor [b]**
**grain**	grain_Mat_I	XANES	-20 to 80	85	16	100	84	16	1.70E-02
**straw**	straw_Mat-III	XANES	-20 to 80	21	78	99	21	79	6.50E-03
	straw_Mat-II	XANES	-20 to 80	21	79	100	21	79	4.88E-03
	straw_Mat-I	XANES	-20 to 80	19	83	103	19	81	6.04E-03
**shoot**	shoot_Flow_I	XANES	-20 to 80	75	25	100	75	25	1.20E-03
		EXAFS	3 to 12	68	33	100	68	33	1.33E-01
**node**	node_Mat_II	XANES	-20 to 80	100	0	100	100	0	2.86E-03
		EXAFS	3 to 11	109	0	109	100	0	2.57E-01
	node_Mat_I	XANES	-20 to 80	100	0	100	100	0	1.64E-03
		EXAFS	3 to 10.5	103	0	103	100	0	5.54E-02
	node_Flow_I	XANES	-20 to 80	100	0	100	100	0	8.40E-04
		EXAFS	3 to 11	106	0	106	100	0	1.38E-01
	node_Flow_II	XANES	-20 to 80	100	0	100	100	0	2.04E-03
**root**	root_Mat_I	XANES	-20 to 80	100	0	100	100	0	7.49E-03
		EXAFS	3 to 11	109	0	109	100	0	3.52E-02
	root_Mat_Ia	XANES	-20 to 80	100	0	100	100	0	2.83E-03
		EXAFS	3 to11	101	0	101	100	0	5.80E-02
	root_Mat_II	XANES	-20 to 80	100	0	100	100	0	1.72E-03
		EXAFS	3 to11.7	93	6	99	94	6	4.11E-02
	root_Flow_I	XANES	-20 to 80	100	0	100	100	0	1.13E-03
		EXAFS	3 to10.7	107	0	107	100	0	3.84E-02
**soil**	soil_Mat_II	XANES	-20 to 80	75	25	100	75	25	1.04E-03
	soil_Mat_I	XANES	-20 to 80	53	47	100	53	47	3.40E-03
	soil_Flow_I	XANES	-20 to 80	38	63	101	38	62	1.90E-03
		EXAFS	3 to 11	34	65	99	34	66	1.27E-01
	soil_Flow_Ia	XANES	-20 to 80	11	88	99	11	89	1.80E-03
	soil_Flow_II	XANES	-20 to 80	31	69	100	31	69	5.70E-03
		EXAFS	3 to 11	16	61	77	21	79	4.60E-01
	soil_initial_I	XANES	-20 to 80	13	88	101	13	87	5.40E-03
		EXAFS	3 to 10	0	99	99	0	100	2.10E-01

Flow: flowering stage. Mat: full maturity stage. I/II/III denote experimental replicates. "a" denotes analytical replicate. Reference compounds used as proxy for Cd-thiol or mixed/ amorph. CdS: Cd-metallothionein, Cd-phytochelatin, Cd-GSH, Cd-cysteine and (Cd, Zn, Fe)S. Reference compounds used as proxy for Cd-O: Cd-organic acid complexes, cd phosphate, Cd phytate, and Cd sorbed on goethite and ferrihydrite.

Table_S 7: Dry weight and micronutrient concentrations in the upper shoot at maturity

compartment	period/ growth stage	DW			Zn						Mn						Ni						Cu					
		dry weight (DW)			concentration			mass			concentration			mass			concentration			mass			concentration			mass		
		g			µg g^{-1}			µg			µg g^{-1}			µg			µg g^{-1}			µg			µg g^{-1}			µg		
		mean	±sd[a]		mean	±sd[a]		mean	±sd[a]		mean	±sd[a]		mean	±sd[a]		mean	±sd[a]		mean	±sd[a]		mean	±sd[a]		mean	±sd[a]	
shoot	maturity	45.0	3.22		40.5	4.15		1575	109		239	14.7		9314	796		0.91	0.14		36	6.1		4.0	0.73		155	24.1	
flag leaves	maturity	1.35	1.05	b	14.5	0.61	c	21.8	15.9	b	245	26.1	b	352	229	b	0.48	0.16	b	0.69	0.47	c	4.23	0.47	ab	6.49	5.02	b
panicle	maturity	2.81	0.69	b	46.7	2.48	a	132	39.3	a	358	21.3	a	1012	295	a	1.38	0.16	a	3.9	1.14	b	3.54	0.65	b	9.92	2.66	b
grains	maturity	4.83	1.01	a	26.9	1.24	b	130	25.6	a	26.1	5.26	c	125	30	c	1.60	0.35	a	7.75	2.35	a	4.78	0.69	a	23.0	5.70	a

[a]sd = standard deviation of the mean of n = 4 experimental replicates. Letters indicate significant differences of the mean determined using ANOVA (non tranformation: DW, Zn mass; 1/x tranformation: Cu conc.; sqrt transformation: Zn conc., Mn conc., Ni conc., Ni mass; log10 transformaition: Mn mass.)
[b]the sd was calculated using error propagation as described in equations 4-5.

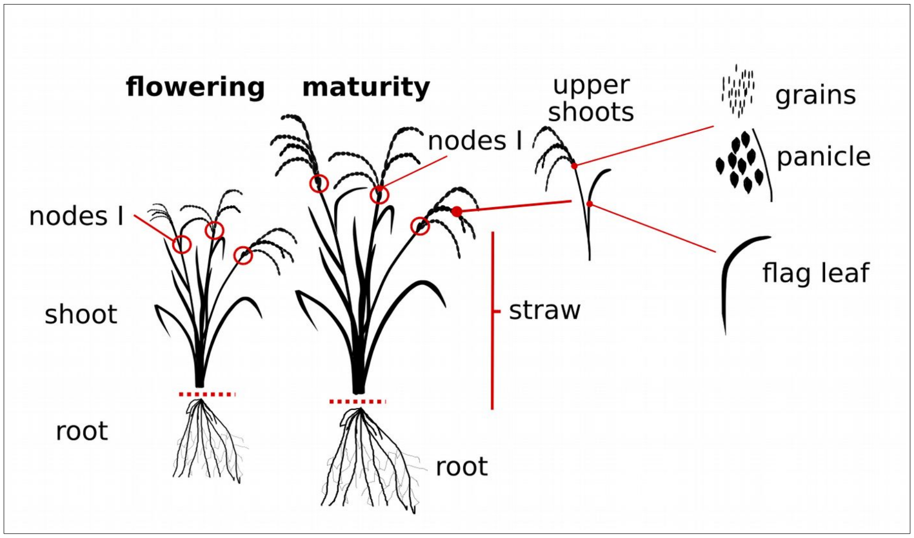

Figure_S 1: Overview rice parts analyzed.

At flowering, the roots were separated from the shoots and then the nodes were separated from the shoot. At maturity, roots were separated form the shoot and the shoot was dissected into nodes, straw, and upper shoot. The latter was further dissected into flag leaves, panicle (rachis and spikelets), and grains. For both growth stages, the nodes sampled are defined as 'node I' according to Yamaji and Ma (2014).
